# A Heterozygous Variant in *HABP2* Causing Increased Risk of Arterial and Venous Thrombosis in a Young Male: Diagnostic and Therapeutic Challenges

**DOI:** 10.1155/carm/1490289

**Published:** 2025-09-25

**Authors:** Salim Al-Busaidi, Nasiba Al-Maqrashi, Khalid Al-Thihli, Bader Al Rawahi, Hatem Al Farhan, Abdullah M. Al Alawi

**Affiliations:** ^1^Department of Medicine, Sultan Qaboos University Hospital, Muscat, Oman; ^2^Oman Medical Specialty Board, Muscat, Oman; ^3^Department of Hematology, Sultan Qaboos University Hospital, Muscat, Oman; ^4^Department of Genetics, Sultan Qaboos University Hospital, Muscat, Oman

**Keywords:** genetic polymorphism, *HABP2*, myocardial infarction, stroke, thrombophilia, thrombosis

## Abstract

Thrombosis is a major clinical issue, affecting venous and arterial circulation, increasing morbidity and mortality. While thrombophilia syndromes are established, new genetic polymorphisms in the *hyaluronan binding protein 2 (HABP2)* gene are not well understood. A 31-year-old man, a smoker, presented with multiple thrombotic events: ST-elevation myocardial infarction (STEMI), ischemic stroke, and left upper limb deep venous thrombosis. On arrival, he experienced ventricular fibrillation, needing resuscitation and percutaneous coronary intervention. During his hospital stay, he developed severe thrombocytopenia, rhabdomyolysis, and acute kidney injury. Extensive thrombophilia workup, including whole exome sequencing, revealed a heterozygous *HABP2* variant linked to thrombotic risk. His condition required a multidisciplinary approach. Genetic findings informed antithrombotic treatment and emphasized family screening. More research is needed on *HABP2* in thrombosis.

## 1. Introduction

Thrombosis is the pathological formation of intravascular blood clots, affecting both venous and arterial circulation, thereby causing disruption to blood flow and leading to consequential clinical outcomes [1]. This pathological process is the basis of a variety of cardiovascular and cerebrovascular diseases, such an ischemic heart disease and its outcome myocardial infarction, ischemic stroke, as well as venous thromboembolism (VTE) [1, 2]. Thrombosis presents a significant global burden, contributing to 25% of global mortality and standing as the leading cause of morbidity and mortality worldwide [2, 3].

Our comprehension of thrombosis throughout history has been shaped by “Virchow's triad,” introduced by Rudolf Virchow in 1856. This triad encapsulates three essential factors: damage to the endothelial lining of blood vessels, a hypercoagulable state, and arterial or venous blood stasis [4]. However, the occurrence of combined arterial and venous thrombotic events in younger individuals, lacking an apparent cause, presents considerable challenges in diagnosing and managing the condition [5].

Genetic variation plays a significant role in determining the risk of thrombosis, even though only a small fraction of patients with thrombosis are discovered to have one of the five established heritable causes of thrombophilia: Factor V Leiden, Prothrombin G20210A mutation, antithrombin (AT) deficiency, protein C (PC) deficiency, and protein S (PS) deficiency [6–8]. Recent advancements in genetic research on thrombosis have revealed significant insights into its underlying mechanisms. Several Genome-wide association studies have identified several thrombosis susceptibility genes, although the biological significance of several variants remains uncertain [9]. Whole exome sequencing (WES) has emerged as a valuable diagnostic tool for assessing patients with thrombosis or inherited thrombophilia. It has proven effective in predicting the involvement of numerous novel genetic variants in thrombosis and expanding the spectrum of susceptibility genes [6, 9].

Here, we present a rare and challenging case involving a 31-year-old male with no prior medical comorbidities apart from smoking. He experienced arterial and venous thrombosis events including myocardial infarction, ischemic stroke, and left upper extremity deep venous thrombosis. A comprehensive, multidisciplinary approach was undertaken to evaluate causes of his extensive thrombosis, including investigations for thrombophilia, atherosclerosis, thromboembolism, medications, substances, vascular and anatomical abnormalities, and systemic disorders. WES revealed a heterozygous variant in *hyaluronan binding protein 2 (HABP2)*, a gene associated with inflammation and recognized as an independent risk factor for both thrombosis and atherosclerosis [10, 11].

## 2. Case Presentation

**Figure 1:**
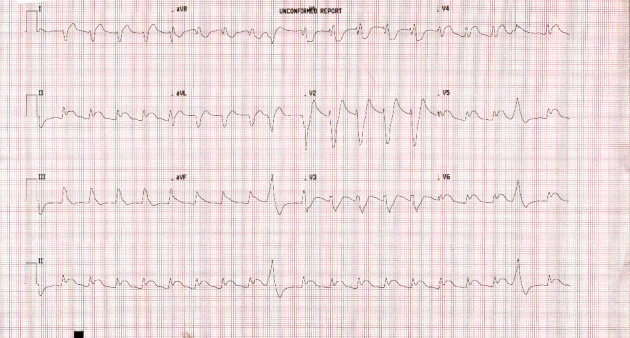
Electrocardiography of the patient on presentation.

A 31-year-old male with long-standing angina and smoking history presented to the emergency on January 10, 2024, with central chest pain associated with shortness of breath and sweating. The patient collapsed on arrival, and cardiopulmonary resuscitation (CPR) was initiated. The initial rhythm was VF for which he received several electrical shocks as well as epinephrine and amiodarone as per advanced cardiac life support (ACLS). Return of spontaneous circulation (ROSC) was achieved after 11 cycles and he required intubation. Initial ECG revealed junctional rhythm with ST-segment elevation in inferolateral leads (Figure 1). A provisional diagnosis of STEMI was made; emergency coronary angiography was done, and it revealed distal thrombus in right coronary artery (RCA) with total occlusion left circumflex (LCX). He underwent treatment with intracoronary and 24-h infusion eptifibatide, and plain old balloon angioplasty (POBA) to LCX and RCA.

The patient had a complex hospital course. He had oliguric AKI and profound unexplained rhabdomyolysis that peaked at a value of CK 580,000 U/L with negative autoimmune workup as well as muscle biopsy. He also developed severe thrombocytopenia consistent with eptifibatide-induced thrombocytopenia (nadir platelet count: 13 × 10^9^/L), which resolved by Day 7. He had myocarditis, liver function derangement, and recurrent hypoglycemia. Acylcarnitine profile in dried blood spots showed elevated free carnitine with low C16 and C18 acylcarnitines suggestive of carnitine palmitoyltransferase 1 (CPT1) deficiency. WES was initiated in view of acylcarnitines' abnormalities and the complex phenotype this patient presented with.

**Figure 2:**
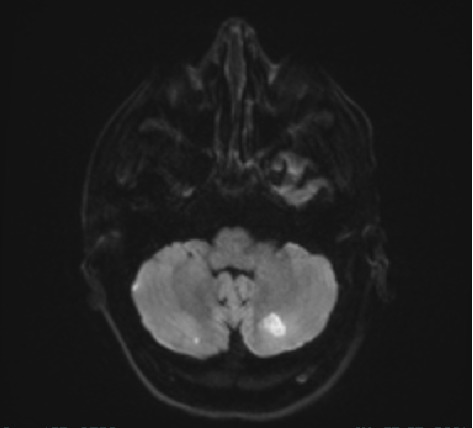
Brain MRI showing multiple area of infracts involving bilateral cerebellum and right frontal.

On Day 8, the patient showed weakness in the right upper and lower limbs (motor scale of 3/5 for both) without any voluntary movement on the left side. The brain MRI showed multifocal ischemic brain infarctions (Figure 2). Later, left upper arm DVT was detected on Day 15, which prompted further evaluation for possible underlying hypercoagulable state.

Coronary angiography excluded atherosclerosis and magnetic resonance imaging of the intracranial arteries was normal. No specific aortic or carotid imaging was done unfortunately. The possibility of embolic sources was entertained but the echocardiogram with bubble studies was noncontributory and there were no any arrhythmias after the admission. Other vascular anatomy appeared normal on multiple imaging studies. Drug-induced thrombosis was considered, but it did not appear that the patient used illicit drugs. Investigations to rule out other systemic disorders with a pan-CT excluding malignancy as well as OGD and colonoscopy with biopsies are normal. Hematological and autoimmune workup is listed in Table 1.

Considering the severe renal impairment, intravenous heparin was given and dual antiplatelet therapy was carefully introduced. The therapy had to be held in view of gastrointestinal bleed, anticoagulation was then cautiously restarted with heparin using low doses and slowly titrating to full-dose infusion. He eventually transitioned to apixaban, starting at 2.5 mg twice daily and later up titrated to 5-mg BID prior to discharge.

The patient was discharged in a good clinical condition with improving neurological status and stable renal function. He was discharged home on apixaban and scheduled for further tight outpatient follow-up. Subsequently, the WES results were negative for CPT1 deficiency but revealed a heterozygous NM_004132.4:c.577_579 delGAC, p.(Asp193del) variant in *HABP2*. The variant was classified as a variant of unknown significance, but it is a very rarely reported in-frame variant in the general population with a frequency of gnomAD: 0.000004 with a likely pathogenic role in thrombophilia.

**Figure 3:**
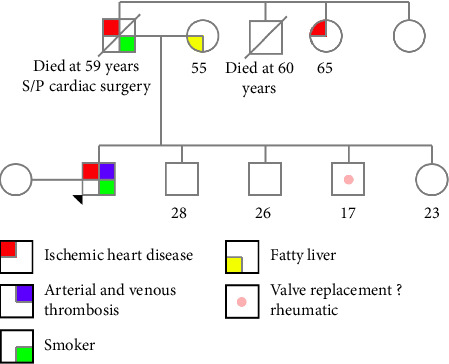
Detailed family pedigree highlighting inherited thrombosis risk.

After discharge, a follow-up included a virtual counseling session to discuss the results of his WES. A hematologist advised on long-term anticoagulation therapy with warfarin, targeting an INR of 2-3, or alternative direct oral anticoagulants (DOACs). Moreover, a comprehensive family history disclosed early-onset coronary artery disease in his father and the sudden death of his uncle, indicating an autosomal-dominant manner of inheritance as well (family lineage; Figure 3). Genetic counseling emphasized the potential risk, with a fifty percent chance of passing the variant to future offspring. Testing of other family members was strongly advised, and targeted mutation testing was encouraged. Given the reported, albeit inconsistently, increased risk of nonmedullary thyroid cancer in association with pathogenic variants in *HABP2*, baseline thyroid ultrasound and vigilance for symptoms were advised. Ongoing follow-up in hematology and genetic counseling clinics was arranged to ensure optimal management and evaluation of his condition along with at-risk family members.

This case highlights important clinical challenges in managing thrombosis associated with rare genetic variants in genes associated with increased risk of thrombophilia like *HABP2*. A multidisciplinary approach and close outpatient follow-up are essential to guide treatment, carefully balancing thrombosis and bleeding risks, while also addressing potential hereditary implications for the patient's family.

## 3. Discussion

This case presents a rare and complex scenario involving a 31-year-old male with no significant medical history aside from smoking, who developed multiple thrombotic events, including myocardial infarction, ischemic stroke, and DVT in the left upper extremity. The unusual combination of both arterial and venous thrombosis in a young, otherwise, healthy individual prompted a thorough and multidisciplinary approach to guide the management.

The patient underwent extensive thrombophilia evaluation which was negative. WES resulted in identification of a heterozygous specifically the p.(Asp193del) variant (NM_004132.4.577_579delGAC) variant in the *HABP2* gene. The in-frame variant is rare in the general population, and no other causative variant was identified to explain the multiple arterial and venous thrombosis in the patient at this young age. The variant was thus considered to be of clinical relevance, particularly that the reported family history of early onset of coronary artery disease is consistent with autosomal dominant inheritance.

The *HABP2* gene, also known as factor VII activating protease (FSAP), encodes a member of the peptidase S1 family of serine proteases. This gene is located on chromosome region10q25–q26, where it spans 35 kb and contains 13 exons. The gene product is initially secreted as a preproprotein by hepatocytes and undergoes proteolytic processing to form a mature heterodimer composed of heavy and light chains. Further autoproteolysis results in the production of smaller, inactive peptides. This extracellular protease binds hyaluronic acid and is believed to play a role in both the coagulation and fibrinolysis systems [12, 13].

Despite the classification as a variant of unknown significance, the role of *HABP2* in thrombosis and atherosclerosis is increasingly recognized due to its involvement in inflammation and vascular integrity [12]. Additionally, it has been associated with acute respiratory distress and malignancies, including nonsmall cell lung carcinoma and familial nonmedullary thyroid cancer [14, 15].

Recent research highlights the connection between *HABP2* mutations and certain diseases. In a large Chinese cohort, the *HABP2* variants rs7923349 and rs932650 were significantly linked to an increased risk of ischemic stroke, with individuals carrying high-risk genotypes showing a notably higher stroke risk (OR: 3.58, 95% CI: 2.62–4.89, and *p* < 0.001) [16]. In familial no-medullary thyroid cancer (FNMTC), the germline *HABP2* G534E mutation was found in seven family members and 4.7% of 423 thyroid cancer patients, suggesting a potential causal role [17]. However, we could not find previous evidence or case reports to associate the *HABP2* NM_004132.4:c.577_579delGAC (p.Asp193del) mutation with any illness.

In our patient, the combination of venous and arterial thrombosis in the absence of clear atherosclerosis and negative conventional thrombophilia workup suggests a genetic predisposition as a compelling mechanism for this unusual presentation. Although the exact role of the *HABP2* gene remains unclear, previous studies have highlighted potential pathogenic pathways [11]. Variants such as the Marburg I and II polymorphisms have been linked to an increased risk of cardiovascular diseases, including stroke, coronary artery disease, and venous thrombosis [18]. These polymorphisms likely contribute to thrombosis by disrupting the balance between fibrinolysis and coagulation, particularly by decoupling the activation of prourokinase and Factor VII [19–21]. Despite the absence of clear evidence of atherosclerosis in our patient, with limited vascular imaging, *HABP2* is known to contribute to atherosclerosis by regulating vascular smooth muscle cell proliferation and promoting plaque instability, ultimately leading to atherothrombosis [22].

There is insufficient evidence in the literature to guide an optimal antithrombotic strategy for patients with inherited genetic thrombophilias, with most sources recommending longer term anticoagulation [23, 24]. In our case, the decision regarding antithrombotic therapy was based on expert opinion, recommending long-term, potentially lifelong anticoagulation with warfarin, targeting an INR of 2-3 to target both arterial and venous thrombosis. Other options, such as DOACs, were considered but deemed less favorable. The potential addition of antiplatelet agents would only be considered if there was evidence of atherosclerosis, particularly if DOACs were selected.

Further clarification of the family history, as outlined in the family pedigree, suggests a possible autosomal dominant inheritance pattern, making the role of this gene mutation more compelling in explaining our patient's complex thrombosis. This pattern of inheritance has also been reported in cases of familial nonmedullary thyroid cancer involving the same gene mutation [15]. For our patient, a baseline thyroid ultrasound was recommended, along with vigilance for symptoms, to ensure early detection of potential thyroid issues. Additionally, targeted mutation testing has been recommended for other family members for segregation analysis given the reported ischemic heart disease and death at a young age with reported ischemic heart disease to assess their risk and guide appropriate monitoring. Unfortunately, none of the family members was available for testing at the time of reporting.

In summary, this case highlights the complexity of managing arterial and venous thrombosis in a young patient with a *HABP2* gene mutation and a negative standard thrombophilia workup. The genetic findings emphasize the need for a tailored, multidisciplinary, and holistic approach. Genetic counseling and family screening are essential due to the likely autosomal dominant inheritance, and ongoing monitoring is crucial to address potential complications and guide long-term management.

## 4. Conclusion

This case report highlights the challenges of diagnosing and managing a complex, stormy disease course triggered by thrombotic events in a young patient with a *HABP2* gene mutation. The identification of this rare genetic variant underscores the importance of considering genetic predispositions in unexplained thrombosis cases. A multidisciplinary approach and personalized care were crucial for effective management. Furthermore, this case emphasizes the need for further research to clarify the role of the *HABP2* mutation in thrombosis and to establish optimal strategies for antithrombotic therapy in such patients.

## Figures and Tables

**Table 1:** Relevant hematological and autoimmune workup.

Protein C functional chromogenic	> 1.5

Protein S functional	1.413

Paroxysmal nocturnal hemoglobinuria (PNH)	Negative for PNH clone on neutrophils and monocytes

Heparin-induced thrombocytopenia (HIT)	Screening for antiheparin-platelet factor 4 (PF4) antibodies (IgG) by rapid test using STic Expert HIT is negative

Myeloproliferative neoplasms (MPNs)	Negative JAK2 (V617F), JAK2 exon12–14, CALR exon 9 MPL exon 10 and BCR/ABL t(9; 22) P210 (CML) not done

Antinuclear antibody (ANA)	1/80

Extractable nuclear antigen (ENA)	Negative

Anti-double-stranded deoxyribonucleic acid antibodies (dsDNA Abs)	< 1

Anti-B 2 glycoprotein 1	< 1

Anticardiolipin antibody	1

Lupus anticoagulant (LA)	Not detected

Antiproteinase 3	Negative

Antimyeloperoxidase	Negative

## Data Availability

The datasets generated and/or analyzed during the current study are available from the corresponding author upon reasonable request.

## Patient Perspective

Patient consent was obtained.
